# Prevalence and Risk Factors for Fall among Rural Elderly: A County-Based Cross-Sectional Survey

**DOI:** 10.1155/2022/8042915

**Published:** 2022-06-27

**Authors:** Hongping Zhang, Yinshaung Zhao, Feng Wei, Mo Han, Jianquan Chen, Songxu Peng, Yukai Du

**Affiliations:** ^1^College of Medicine and Health Science, Wuhan Polytechnic University, Wuhan 430023, China; ^2^Centers for Disease Prevention & Control of Huangpi District of Wuhan, Wuhan 430300, China; ^3^Department of Disease Control, Health and Family Planning Commission of Huangpi District of Wuhan, Wuhan 430300, China; ^4^Department of Maternal and Child Health, Xiangya School of Public Health, Xiangya School of Medicine, Central South University, Changsha 410008, China; ^5^Department of Social Medicine and Health Management, School of Public Health, Tongji Medical College, Huazhong University of Science & Technology, Wuhan 430030, China

## Abstract

**Aim:**

The aim of the study was to provide evidence for the prevention and reduction of falls in the elderly living in rural areas by analyzing epidemiological data of falls among the rural older people (>65 years old) and identifying the risk and protective factors.

**Methods:**

This study analyzed the sociodemographic characteristics, living environment, lifestyle, chronic disease condition, mental health, activities of daily living (ADL), and detailed information of falls of 3752 rural elderly. Rank tests, chi-square tests, and binary logistic regression were used for data analysis.

**Results:**

The prevalence of falls was 30.0%, and the 75–84-years age group had the highest fall rate (18.8%). According to the binary logistic regression analysis, six variables, including roughage intake frequency, age, gender, cane use, floor tiles, and IADL, were involved in the fall patterns. Low roughage intake (OR = 2.48, 95% CI 1.24–4.97), female gender (OR = 2.12, 95% CI 1.48–3.05), the use of a cane (OR = 2.11, 95% CI 1.08–4.10), and medium IADL (OR = 2.02, 95% CI 1.89–2.32) were the top four risk factors.

**Conclusion:**

The fall in the rural elderly was mainly due to the poor living and working conditions. Routine fall assessment could address several preventable risk factors to reduce the prevalence and mitigate the harm of falls.

## 1. Introduction

Fall injuries in older adults are becoming a major public health concern with the aging population. Falls are the second leading cause of unintentional injury deaths and the 13th leading cause of years lived with disability (YLD) globally [1]. Approximately 28–35% of people aged ≥65 years fall at least once a year [2]. Falls in the elderly are the most common cause of immobility, mortality, hospitalization, loss of independence, poor quality of life, and early entry to long-term care facilities [3]. Thus, developing an effective fall prevention strategy is imperative to mitigate the harm of falls in the elderly [2].

Several studies have investigated the current state and risk factors of falls. Age is one of the risk factors for falls in the elderly [4]. Fall-related injuries rise with age, with an increased risk of fracture, especially hip, in >50% of the cases [5]. Strikingly, falls among the elderly females are higher than in elderly males [6]; females >70 years old are prone to bone fractures [7]. Some chronic diseases, such as orthostatic hypotension, lumbar spondylosis, blood pressure, diabetes, cataract, musculoskeletal diseases, and urinary incontinence (UI), are risk factors for falls in the elderly [8, 9]. Open-angle glaucoma and severe bilateral visual deficits add risks to falls in the elderly [10, 11]. Also, individuals with depression are prone to fall [6, 8]. The use of medications also explains the increased rate of fall [12]. Also, severe or extreme problems in sleeping, alcohol abuse, a fall history, and the fear of falling are the risk factor for falls [13]. The fall risk among the elderly is closely related to the activities of daily living (ADL) capability, obesity, physical activity habits, poor living conditions, and environmental factors [14].

The fall of the elderly is still a major challenge worldwide, but most studies focus on urban-dwelling older adults. The elderly living in rural areas may have a higher risk of falls than those in the urban areas because most of them are farmers with a low educational level, poor economic conditions [15], and an increased risk of developing musculoskeletal diseases due to aging and agricultural lifestyle. Compared to most previous studies conducted in the community or hospitalized patients [7, 16], the reports about the conditions and risk factors of falls in rural elderly are limited [17].

The data on falls in rural areas differed markedly, and some were contradictory. Some studies reported that rural areas confer a significant risk of falls [6], and the injuries observed in rural areas are severe [18]. Another study reported that falls were lower in rural areas than in urban areas [19, 20]. A review on falls in rural elderly reported that there were no significant differences in fall hospitalization, mortality, or injuries sustained among rural, remote, and major city residents [21]. Some studies focused on urban-dwelling elderly reporting that the frequency of falls increases with age, which is not observed in rural areas. A few studies reported that age is not a risk factor for falls, and younger elderly (60–69 years old) suffered more falls and exhibited statistically significant factors related to falls [22], while another report deemed this feature more probable in the 70–79-year-old age group [23]. Tripping was the most common cause of a fall in the community [24]. Commonly identified home hazards, which increased the fall risks in rural Victorian homes, were home access, i.e., movement around the house and the bathroom [15]. Specific environmental home hazards relevant to the rural setting differ from those in the regional or metropolitan settings; these include large houses, outdoor toilets, and a variety of ground surfaces [15].

### 1.1. Hypotheses

Risk factors associated with falls can be divided into intrinsic and extrinsic factors [15]. A fall does not occur because of one factor alone but rather when physical, psychological, social, and environmental factors work together. The rural elderly have a different lifestyle and living environments; hence, the risk factors associated with the falls of the elderly in rural areas are different from that of urban dwellers. Thus, it is crucial to identify the risk factors for falls by considering the individual characteristics of the elderly in rural areas and their living environments.

## 2. Methods

### 2.1. Study Design

This cross-sectional study conducted a cluster sampling of 3752 rural elderly (>65 years old) in the Huangpi county of Wuhan City, Hubei Province, China. The study aimed to provide evidence for the prevention and reduction of falls in the rural elderly population by analyzing the epidemiological data, such as sociodemographic characteristics, economic status, living environment, the prevalence of chronic diseases, mental condition, ADL, and IADL of the elderly of fall and identifying the involved risk and protective factors [25].

### 2.2. Sample Size

The sample size formula was *N* = *Z*_a/2_^2^ × *π*(1-*π*)/*δ*^2^ = 576 (the sample size was obtained to achieve a confidence level of 95%, with a 5% margin of error and 50% prevalence of elderly falls). Based on the different geographical locations (mountainous areas and flatlands) and economic levels (poor, medium, and good) (data on rural residents' per capita income were from the *XXX district statistical yearbook* (2015)), the sample size should be 576 × 2 × 3 = 3456. Considering the possibility of invalid responses, the actual sample size was increased by 5%, *N* = 3456 × (1 + 5%) = 3628.8; thus, 3630 individuals were selected.

### 2.3. Inclusion and Exclusion Criteria

Two townships were selected from each economic level (poor, medium, and good) and geographical locations (mountainous areas and flatlands). Then, two villages were selected from each township. Finally, all the elderly (≥65 years old) who lived in the 24 villages of Huangpi county comprised the study population. If the elderly had any language or cognitive impairment, they could be replaced by asking their spouse or family members. The exclusion criterion was the absence of a registered permanent rural address or residing in the specific village for <6 months.

### 2.4. Procedure

The investigator team was composed of 16 postgraduates from the School of Public Health of Huazhong University of Science and Technology. We set up a team leader to be responsible for the overall planning and coordination of the site. All the investigators received training for the survey. The training contents included clarifying the purpose and significance of this baseline survey, being familiar with the whole process of the baseline survey, clarifying the survey object, content, purpose, and method of each questionnaire becoming familiar with the outline of the qualitative interview, clarifying the contents of the individual interview or group discussion, knowing the accident handling measures and matters needing attention in the investigation, and understanding the responsibilities to ensure the quality of investigation.

Currently, the government has promoted free medical examinations for the elderly. Thus, after the elderly underwent the free medical examination in the township hospitals, we asked them to complete the questionnaires. The evaluation of the health status of the elderly (height, weight, blood pressure, and bone density) was carried out immediately by the personnel using the equipment provided by Huangpi District Health and Family Planning Commission. Those who did not partake in the medical examination were evaluated at their homes or village public area and asked to complete the questionnaires. For those elderly who were not in the village, the questionnaires were completed by telephone or the village doctor.

### 2.5. Measures

A self-designed questionnaire was administered for cluster sampling to investigate the sociodemographic characteristics, economic status, living environment, offspring, the prevalence of chronic diseases, mental health, ADL, and IADL of the elderly.

The questionnaire was composed of the following seven sections from Part A–G: Part A showed the personal social demographic characteristics, such as age, nationality, gender, education, occupation, marital status, offspring condition of the elderly, and economic situation. Part B showed the information about daily lifestyles, such as dietary and exercise habits. Part C covered detailed information on the treatment, the nature of falls, and associated costs. Part D gathered information about the living environment, including illumination, stairs, temperature, ground, and toilet design. Part E collected information on the chronic disease conditions of the older individuals and their use of health services. Part F showed that K10 was used to check the mental state of the elderly. Part G included the ADL (Barthel index (BI)) and IADL scales. The Lawton IADL questionnaire evaluated seven IADL functions [24].

To ensure the quality of the collected data, this study was first conducted as a small-scale pilot questionnaire-based survey, encompassing 150 samples from one village. This pilot study was also used to train the research staff in managing the data collection and entry. After this pretest, the questionnaire was modified.

The data from the final questionnaires were analyzed statistically. Based on Cronbach's alpha and factor analysis, the questionnaire has high validity and reliability: Cronbach's alpha reliability coefficient was 0.721, and KMO validity coefficient was 0.739 on Part B; Cronbach's alpha reliability coefficient was 0.869, and KMO validity coefficient was 0.780 on Part D; Cronbach's alpha reliability coefficient was 0.940, and KMO validity coefficient was 0.935 on Part F; Cronbach's alpha reliability coefficient was 0.939, and KMO validity coefficient was 0.932 on Part G. These results confirmed the validity and reliability of the questionnaire.

### 2.6. Statistical Analyses

The data were entered independently by two investigators using EpiData3.1. SPSS version 21.0 was used for statistical analysis. Descriptive analysis, chi-square test, and binary logistic regression analysis were performed.

## 3. Results

### 3.1. General Description of the Investigation

A total of 3,900 questionnaires were distributed, of which 3,752 completed questionnaires were received, with a response rate of 96.2%. The database covered 2,079 females (55.4%) and 1,673 males (44.6%). The average age of the cohort was 72.74 ± 6.44 (range: 65–100) years. In this survey, 64.8% of the population was 65–74 years old, 29.9% were 75–84 years old, and 5.3% were 85 years old (Table 1). About 68.2% were married and living with a spouse, 39.3% obtained primary education, and 38.5% did not have any formal education. About 74.8% were farmers and not gainfully employed. Among the participants, 68.9% earned <5,000 RMB, and 38.0% had an economic dependence on government assistance. About 95.6% have children, and 35.7% were visited by their son or daughter.

During the 12 months of this survey, encompassing 3,752 responders aged >65 years old, 610 victims reported 1,124 fall injuries caused by the fall. The observed fall prevalence was 30.0% in this study. About 45.5% of falls occurred in summer, and 23.8% occurred in August. The top three most common reasons for falls are slippery ground (24.1%), leg weakness (21.1%), and obstacles in the corridors (20.6%). The lower limb (34.5%) and upper limb (29.4%) were the most commonly reported anatomical sites of fall injuries. The most common fractures were upper (41.0%) and lower extremities (32.3%). The top three most common pathological types of injuries were bruises, fractures, and sprains. The top three most common places in which falls occurred were the living room (32.7%), on the road (20.6%), and the workplace (20.1%). Walking (54.6%) was the most common activity when a fall happened, followed by working (17.1%) (Table 2).

### 3.2. Sex- and Age-Based Fall Patterns

A statistically significant correlation was established between fall and age/sex. The 75–84-year-old age group had the highest fall rate (18.8%), followed by the 65–74-year-old age group (15.3%), while ≥85-year-old age group had the lowest rate (13.1%) (*P* < 0.05). The fall prevalence in the female responders was significantly higher (19.4%) than that of the male responders (12.3%) (*P* < 0.01) (Table 1). Although walking and working were the two main reasons for the fall, it is more likely for males to experience working (22.8% *vs*. 16.4%) (*P* < 0.05) and females to experience walking (64.8% *vs*. 49.2%) (Table 2).

### 3.3. Treatments and Costs

In this study, 29.6% (*n* = 212) of the fall victims did not receive any treatment, 21.6% (*n* = 122) were self-medicated, and 48.8% (*n* = 276) of the study population sought medical help (including 10.6% in village clinics, 16.3% in town hospitals, 15.6% in district hospitals, 3.8% in city-level hospitals or above, and 2.5% in private clinic). The total cost of 1,124 injuries was 1,213,650 RMB; 121 people were hospitalized for 1882 days with an expenditure of 95,553 RMB. Due to falls, the hospitalization rate of men was higher than that of elderly women (27.7% *vs*. 19.5%, *P* < 0.05), and more elderly males suffered limited physical activity than females (37.4% *vs*. 32.3%, *P* < 0.05) (Table 3).

### 3.4. Risk Factors

The current study identified 30 factors related to falls by chi-square test: gender, age, mental state, ADL, IADL, boon density, marital status, education level, income level, source of income, floor tiles, height of stairs, domicile near road, fresh fruit intake frequency, meat intake frequency, roughage intake frequency, cane use, eyesight, sleep duration, hypertension, diabetes, heart disease, tumor, stroke, arthritis, cervical spondylosis, hepatocirrhosis, cataract, and the number of medicines. Then, we conducted a binary logistic regression analysis on these factors to assess their association with the occurrence of falls as the dependent variable. According to this analysis, six variables, including gender, age, floor tiles, cane use, roughage intake frequency, and IADL, were involved in the fall patterns (Table 4).

Older females were 2.12 times (odds ratio (OR) = 2.12, 95% confidence interval (CI): 1.48 : 3.05) more prone to fall compared to older males. The odds of fall among individuals aged 75–84 years were 1.20 times higher than that of the 65–74-year-old group (OR = 1.20, 95% CI: 1.12–1.75). Elderly aged ≥85 years old had a 0.04 greater risk of fall (OR: 0.04, 95% CI: 0.00–0.49) than those in the 65–74-year-old age group. The oldest individuals displayed the lowest odds of falls. People with slippery floors had a 13% higher possibility of falling (OR 1.13, 95% CI: 0.69–1.84), and people with no floor (with earthen or cement floor) had a 85% higher chance of sustaining a fall (OR = 1.85, 95% CI: 1.23–2.77) compared to older adults with a nonslippery floor. Individuals who use a cane were more than 2.11 times to suffer a fall (OR = 2.11, 95% CI: 1.08–4.10) compared to individuals with no cane. In this study, the risk increased as roughage intake frequency declined. Compared to people scoring “very well” for IADL, older adults with “good” IADL had a 26% higher probability of fall (OR = 1.26, 95% CI: 1.01–1.53), “medium” IADL was 2.63 times higher probability (OR = 2.63, 95% CI: 1.89–3.32), and those who had “poor” IADL had a 16% higher chance of sustaining a fall (OR = 1.16, 95% CI: 1.06–1.45).

## 4. Discussion

The prevalence of falls was 30.0% in this elderly rural population, which is higher than the fall prevalence 36.6/1,000 in rural Bangladesh [26], 11.4% in Ecuador [27], 27.9% in Brazil [22], but lower than 49.9% in Riyadh [28].

The most common places where falls occurred were outdoors, such as roads and the workplace; the most common activities when falls occurred were walking and working; the most common reason that led to fall was slippery ground; the most common reason for falls was summer and specifically autumn, as plenty of rain in summer and farm work in autumn led to the slippery ground. Therefore, the main reason for falls among the rural elderly was work and poor living and working conditions.

The comparison of fall information between males and females did not detect any differences except in the activity when the fall occurred. Typically, males fell easily at work, while females were more likely to fall while walking.

Multivariate logistic regression analyzed six variables: age, floor tiles, gender, use of a cane, roughage intake frequency, and IADL.

Older females were more likely to suffer falls than older males, consistent with other studies. In this study, females were more prone to experience falls, but males had higher rates of hospitalization, and older males suffered limited physical activity than females.

A large number of studies showed that aging is a risk factor for falls. However, the current study found that aging is not a nonlinear correlation risk factor for falls, similar to some previous reports [22, 23]. This study showed that the tendency to fall increases with age before 85 years, and then the risk declines at >85 years of age. The age-related decline in stepover ability and the overestimation or decreased underestimation of this ability might raise the potential risk of falls [29]. The rural elderly need to work in the field daily; thus, 75–84-year-old age group had an age-related physical decline and tended to overestimate their stepover ability; they easily sustained the fall when they stepped out for work. Individuals >85 years old may correctly estimate their ability and reduce their work and avoid falls. Hence, age must be considered in light of individual functions. The older individuals without floors in their homes could easily bear falls compared to people with finished anti-slip floor tiles or those with slippery floor tiles. A house without a floor indicates uneven mud or concrete floors in their homes, which might be uneven ground or more slippery, causing the rural elderly to fall. The unfinished floor in the house might be due to low economic conditions. The reason why the financial situation was not a contributing factor for the elderly in rural areas might be because the people were unwilling to reveal their real economic situation to others. Thus, improving the living environment and economic conditions that increase the risk of residential falls may effectively reduce the incidence of injuries. It is likely that people who use canes to sustain fall and those who use crutches cannot walk and balance freely, increasing the possibility of falls.

Another study showed that a higher ADL was associated with fall [14]. However, in this study, the fall risk increased as the IADL score declined, while the fall risk is not related to ADL capability. This phenomenon could be attributed to the fact that the rural elderly usually work outdoors and may retain a high ADL. If the ADL functions were declined, the elderly would not work outdoors, which would reduce the chance of falls. The reason may be similar to that for the >85-year-old group; people with a declined ADL score may correctly estimate their ability and then reduce their work and avoid falls. Therefore, binary logistic regression analysis was used to evaluate the IADL scale to enter the fall patterns.

Interestingly, we found that daily intake of roughage is a protective factor in preventing falls. Another study showed that roughage intake could reduce the rate of some chronic diseases, such as chronic kidney disease and active Crohn's disease [30]. Roughage is negatively associated with fasting insulin levels in males and females [31]. Taken together, there may be a correlation between roughage intake and chronic diseases, which could make the elderly prone to experiencing falls. Therefore, additional studies are required to explore the link between falls and normal roughage intake.

## 5. Conclusions

In this study, we found that falls are closely associated with gender, age, floor tiles, cane use, roughage intake frequency, and IADL. We also deduced that the reason for falls in the rural elderly was mainly poor living and working conditions. Several preventable risk elements could be addressed by routine fall evaluation to reduce the prevalence and mitigate the harm of falls.

### 5.1. Limitations of the Study

1. The sampling survey only considered the rural elderly in one county, which would introduce a certain degree of bias.
2. The study only used a cross-sectional survey. Interventions and analysis of the outcomes in the next step would be beneficial.

## Figures and Tables

**Table 1:** Comparison of the rate of falls between the genders and ages in a rural elderly population.

	Total (N, %)	Fall (N, %)		*P*-value
		Yes	No	
Total	3752	610	3142	
Age (y)				
65–74	2431 (64.8)	373 (15.3%)	2058 (84.7%)	0.016
75–84	1122 (29.9)	211 (18.8%)	911 (81.2%)	
≥85	199 (5.3)	26 (13.1%)	173 (86.9%)	
Sex				
Male	1673 (44.6)	206 (12.3%)	1467 (87.7%)	≤0.001
Female	2079 (55.4)	404 (19.4%)	1675 (80.6%)	

^*∗*^ *P* < 0.05; ^*∗∗*^*P* < 0.01.

**Table 2:** Description of the reason, anatomical site, pathological type, and location of falls and comparison between the genders in a rural elderly population.

	Total		Sex				*P*-value
			Male		Female		
	N	%	N	%	N	%	
Total	3752		1673	44.6	2079	55.4	
*Season*							
Spring	128	17.3	38	19.3	90	23.7	0.148
Summer	337	45.5	110	55.8	227	59.7	
Autumn	176	23.8	49	24.9	127	33.4	
Winter	100	13.5	35	17.8	65	17.1	

*Fall reason*							
Slippery ground	165	24.1	56	32.6	109	29.8	0.083a
Obstacles in corridors	141	20.6	34	19.8	107	29.2	
Unsuitable clothes and shoes	5	0.7	2	1.2	3	0.8	
Poor eyesight	35	5.1	15	8.7	20	5.5	
Leg weakness	144	21.1	40	23.3	104	28.4	
Dizzy	127	18.6	39	22.7	88	24.0	
No bedside rail restraint	8	1.2	2	1.2	6	1.6	
Trance	45	6.6	9	5.2	36	9.8	
Other	14	2.0	7	4.1	7	1.9	

*Anatomical site of fall*							
Head and neck	70	10.8	24	13.3	46	12.7	0.612a
Spinal bone and back	48	7.4	15	8.3	33	9.1	
Upper extremities	190	29.4	52	28.9	138	38.1	
Lower extremities	223	34.5	80	44.4	143	39.5	
Chest	26	4.0	9	5.0	17	4.7	
Abdomen	12	1.9	3	1.7	9	2.5	
Pelvis	21	3.2	9	5.0	12	3.3	
Buttock	54	8.3	17	9.4	37	10.2	
Other anatomical sites	3	0.5	1	0.6	2	0.6	

*Pathological type of fall*							
Bruise	274	46.8	98	56.3	176	49.4	0.506a
Twist	124	21.2	39	22.4	85	23.9	
Dislocation	15	2.6	3	1.7	12	3.4	
Fracture	133	22.7	38	21.8	95	26.7	
Open (penetrating trauma)	11	1.9	3	1.7	8	2.2	
Visceral injury	3	0.5	0	0.0	3	0.8	
Brain dysfunction	10	1.7	2	1.1	8	2.2	
Crush	14	2.4	6	3.4	8	2.2	
Other	2	0.3	1	0.6	1	0.3	

*Fall place*							
Living room	218	32.7	64	31.5	154	39.3	0.421a
Bathroom	41	6.2	14	6.9	27	6.9	
In the yard	116	17.4	36	17.7	80	20.4	
On the road	137	20.6	54	26.6	83	21.2	
Workplace (farmland)	134	20.1	48	23.6	86	21.9	
Public place	18	2.7	5	2.5	13	3.3	
Other places	2	0.3	1	0.5	1	0.3	

*Activity*							
Taking a shower	23	3.6	10	5.1	13	3.4	<0.05
Going to the toilet	37	5.9	12	6.1	25	6.5	
Going to bed or out of bed	43	6.8	19	9.6	24	6.3	
Walking	345	54.6	97	49.2	248	64.8	
Exercising	32	5.1	20	10.2	12	3.1	
Working	108	17.1	45	22.8	63	16.4	
Going on a ride	15	2.4	6	3.0	9	2.3	
Other	29	4.6	8	4.1	21	5.5	

*Anatomical site of fracture*							
Skull	10	5.1	3	5.5	7	5.4	0.282a
Vertebrae	21	10.8	7	12.7	14	10.8	
Collum femoris	2	1.0	1	1.8	1	0.8	
Upper limbs	80	41.0	18	32.7	62	47.7	
Lower limbs	63	32.3	20	36.4	43	33.1	
Pelvis	13	6.7	3	5.5	10	7.7	
Ribs	6	3.1	4	7.3	2	1.5	

^*∗*^ *P* < 0.05; % = rate; “a” is Fisher's exact test.

**Table 3:** Comparison of the treatments and outcomes of unintentional injuries between the genders in a rural elderly population.

`	Total		Sex				*P*-value
			Male		Female		
	N	%	N	%	N	%	
*Treatment*							
No treatment	212	29.6	67	32.6	113	28	0.403
Self-treatment	122	21.6	37	18.1	95	23.4	
Town hospitals	92	16.3	34	16.6	65	16.1	
District hospitals	88	15.6	35	17.1	60	14.8	
Village clinics	60	10.6	18	8.8	47	11.6	

*City-level hospitals*							
Or above	22	3.9	11	5.2	13	3.2	
Private clinic	14	2.5	3	1.6	12	3	

*Hospitalization*							
Yes	136	22.3	57	27.7	79	19.5	0.029^*∗*^
No	474	77.7	149	72.3	325	80.5	

*Fall severity*							
No adverse reaction	224	36.7	94	45.5	129	32	<0.01
Discomfort	184	30.1	41	19.9	144	35.7	
Physical activity limited	203	33.2	71	34.7	130	32.3	

*Types of payments*							
New type of rural cooperative medical system	321	52.6	105	51.1	215	53.3	0.289a
Self-paying	235	38.5	79	38.2	157	38.8	
Urban residents' health insurance	40	6.5	13	6.1	27	6.7	
Free medical service	13	2.1	8	3.8	5	1.2	
Commercial insurance	2	0.3	2	0.8	0	0	

*Limited function*							
Without limitations	228	37.3	87	42	141	34.8	0.300
1–14 days	138	22.7	37	18.2	101	25	
15–30 days	52	8.6	16	8	36	8.9	
1–3 m	87	14.3	33	15.9	54	13.4	
>3 m	104	17.1	33	15.9	72	17.9	

**Table 4:** Binary logistic regression analysis results: risk factors for fall.

	All injuries		
	Odds ratio	95% CI	*P*-value
Female	2.12	1.48–3.05	≤0.001
Age (years)			
65–74	1.00		
75–84	1.20	1.12–1.75	0.336
≥85	0.04	0.00–0.49	0.011
Floor			
Nonslippery floor	1.00		Reference
Slippery floor	1.13	0.69–1.84	0.636
Without floor	1.85	1.23–2.77	0.003
Using a cane	2.11	1.08–4.10	0.028
Roughage intake frequency			
Daily	1.00		Reference
Often	2.14	1.07–4.31	0.032
Occasionally	2.26	1.12–4.60	0.024
Hardly	2.48	1.24–4.97	0.011
IADL			
Very well	1.00		Reference
Good	1.26	1.01–1.53	0.734
Medium	2.02	1.89–2.32	≤0.001
Poor	1.16	1.06–1.45	0.848

## Data Availability

The datasets used and/or analyzed during the current study are available from the corresponding author on reasonable request.

## Supplementary Materials

Supplementary Materials Graphical Table of Contents. Supplemental table: Factors associated with falls by chi-square test.

## References

[1] Collaborators GMaCoD (2016). Global, regional, and national life expectancy, all-cause mortality, and cause-specific mortality for 249 causes of death, 1980-2015: a systematic analysis for the Global Burden of Disease Study 2015. *Lancet (London, England)*.

[2] Hu X., Qu X. (2016). Pre-impact fall detection. *BioMedical Engineering Online*.

[3] Jeon M. Y., Jeong H., Petrofsky J., Lee H., Yim J. (2014). Effects of a randomized controlled recurrent fall prevention program on risk factors for falls in frail elderly living at home in rural communities. *Medical Science Monitor: International Medical Journal of Experimental and Clinical Research*.

[4] Poulose N., Raju R. (2014). Aging and injury: alterations in cellular energetics and organ function. *Aging and disease*.

[5] Rimland J. M., Abraha I., Dell’Aquila G. (2016). Effectiveness of non-pharmacological interventions to prevent falls in older people: a systematic overview. The senator project ontop series. *PLoS One*.

[6] Stewart Williams J., Kowal P., Kowal P. (2015). Prevalence, risk factors and disability associated with fall-related injury in older adults in low- and middle-incomecountries: results from the WHO Study on global AGEing and adult health (SAGE). *BMC Medicine*.

[7] Jagnoor J., Suraweera W., Keay L., Ivers R. Q., Thakur J., Jha P. (2012). Unintentional injury mortality in India, 2005: nationally representative mortality survey of 1.1 million homes. *BMC Public Health*.

[8] Ouyang P., Sun W. (2018). The association between depressive symptoms and fall accidents among middle-aged and elderly people in China. *Environmental Health and Preventive Medicine*.

[9] Palagyi A., McCluskey P., White A. (2016). While we waited: incidence and predictors of falls in older adults with cataract. *Investigative Opthalmology & Visual Science*.

[10] Lee D. J., Gómez-Marín O., Lam B. L., Zheng D. D. (2003). Visual impairment and unintentional injury mortality: the national health interview survey 1986-1994. *American Journal of Ophthalmology*.

[11] Tanabe S., Yuki K., Ozeki N., Shiba D., Tsubota K. (2012). The association between primary open-angle glaucoma and fall: an observational study. *Clinical Ophthalmology*.

[12] Söderberg K. C., Laflamme L., Möller J. (2013). Newly initiated opioid treatment and the risk of fall-related injuries. A nationwide, register-based, case-crossover study in Sweden. *CNS Drugs*.

[13] Lee S., Oh E., Hong G. S. (2018). Comparison of factors associated with fear of falling between older adults with and without a fall history. *International Journal of Environmental Research and Public Health*.

[14] Maneeprom N., Taneepanichskul S., Panza A. (2018). Fall among physically active elderly in senior housings, Bangkok, Thailand: situations and perceptions. *Clinical Interventions in Aging*.

[15] Yoo J. S., Kim C. G., Yim J., Jeon M. Y. (2016). Factors influencing falls in the frail elderly individuals in urban and rural areas. *Aging-Clinical & Experimental Research*.

[16] Xue C. B., Xiang Y. U., Liu X. Q., Zhu J. G., Zhang D. Z. (2010). Non-fatal Injuries and Risk Factors Among Older Adults in Jiangsu Province. *Chinese Journal of Disease Control & Prevention*.

[17] Saveman B. I., Björnstig U. (2011). Unintentional injuries among older adults in northern Sweden--a one-year population-based study. *Scandinavian Journal of Caring Sciences*.

[18] Burrows S., Auger N., Gamache P., Hamel D. (2013). Leading causes of unintentional injury and suicide mortality in Canadian adults across the urban-rural continuum. *Public Health Reports*.

[19] Li F. F., Zhou D. D., Ye Z. F. (2019). Epidemiologic characteristics of fall in the elderly in urban and rural areas in Shanghai. *Zhonghua liu xing bing xue za zhi = Zhonghua liuxingbingxue zazhi*.

[20] Chen X., Lin Z., Gao R., Yang Y., Li L. (2021). Prevalence and associated factors of falls among older adults between urban and rural areas of shantou city, China. *International Journal of Environmental Research and Public Health*.

[21] Boehm J., Franklin R. C., King J. C. (2014). Falls in rural and remote community dwelling older adults: a review of the literature. *Australian Journal of Rural Health*.

[22] Santos F. D., Lange C., Llano P. M. P. (2019). Falls of elderly people living in rural areas: prevalence and associated factors. *Revista Brasileira de Enfermagem*.

[23] Park D., Jo H., Yoon C. H., Lee E. S., Oh M.-K., Lee C. H. (2019). Fall risk assessment of rural elderly population in korea. *Annals of rehabilitation medicine*.

[24] Wang H., Coppola M., Robinson R. D. (2013). Geriatric trauma patients with cervical spine fractures due to ground level fall: five years experience in a level one trauma center. *Journal of Clinical Medicine and Research*.

[25] Zhang H., Wei F., Han M., Chen J., Peng S., Du Y. (2017). Risk factors for unintentional injuries among the rural elderly: a county-based cross-sectional survey. *Scientific Reports*.

[26] Wadhwaniya S., Alonge O., Ul Baset M. K., Chowdhury S., Bhuiyan A. A., Hyder A. A. (2017). Epidemiology of fall injury in rural Bangladesh. *International Journal of Environmental Research and Public Health*.

[27] Orces C. H. (2014). Prevalence and determinants of fall-related injuries among older adults in Ecuador. *Current gerontology and geriatrics research*.

[28] Almegbel F. Y., Alotaibi I. M., Alhusain F. A. (2018). Period prevalence, risk factors and consequent injuries of falling among the Saudi elderly living in Riyadh, Saudi Arabia: a cross-sectional study. *BMJ Open*.

[29] Sakurai R., Fujiwara Y., Ishihara M., Higuchi T., Uchida H., Imanaka K. (2013). Age-related self-overestimation of step-over ability in healthy older adults and its relationship to fall risk. *BMC Geriatrics*.

[30] Zhao M., Zhu W., Gong J. (2015). Dietary fiber intake is associated with increased colonic mucosal GPR43+ polymorphonuclear infiltration in active crohn’s disease. *Nutrients*.

[31] Byrd-Williams C. E., Strother M. L., Kelly L. A., Huang T. T. K. (2009). Dietary fiber and associations with adiposity and fasting insulin among college students with plausible dietary reports. *Nutrition*.

