# Supplementary material for: Prevalence and Risk Factors for Fall among Rural Elderly: A County-Based Cross-Sectional Survey
Source: Int J Clin Pract. 2022 Jun 27;2022:8042915. doi: 10.1155/2022/8042915 (PMC9252676; doi:10.1155/2022/8042915)
Supplement: Supplementary Materials — Graphical Table of Contents. Supplemental table: Factors associated with falls by chi-square test. [file 8042915.f1.zip › 8042915.f1/Graphical Table of Contents (1).docx]

**Prevalence and Risk Factors for Fall among Rural Elderly:**

**A county-based Cross-sectional Survey**

Hongping Zhang^1*^, Yinshaung Zhao^1^, Feng Wei^2^, Mo Han^2^,

Jianquan Chen^3^, Songxu Peng^4^, Yukai Du^5^


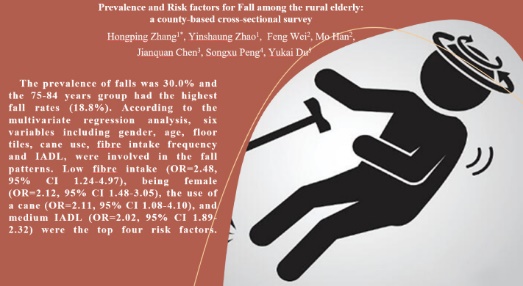


The prevalence of falls was 30.0% and the 75-84 years group had the highest fall rates (18.8%). According to the multivariate regression analysis, six variables including gender, age, floor tiles, cane use, fibre intake frequency and IADL, were involved in the fall patterns. Low fibre intake (OR=2.48, 95% CI 1.24-4.97), being female (OR=2.12, 95% CI 1.48-3.05), the use of a cane (OR=2.11, 95% CI 1.08-4.10), and medium IADL (OR=2.02, 95% CI 1.89-2.32) were the top four risk factors.
