# Supplementary material for: Prevalence and Risk Factors for Fall among Rural Elderly: A County-Based Cross-Sectional Survey
Source: Int J Clin Pract. 2022 Jun 27;2022:8042915. doi: 10.1155/2022/8042915 (PMC9252676; doi:10.1155/2022/8042915)
Supplement: Supplementary Materials — Graphical Table of Contents. Supplemental table: Factors associated with falls by chi-square test. [file 8042915.f1.zip › 8042915.f1/Supplemental table (1).docx]

| **Supplemental Table**  Factors associated with falls by Chi-square test   \|  \| \| Fall (N, %) \| \|  \|  \| \| --- \| --- \| --- \| --- \| --- \| --- \| \|  \|  \| no \| yes \| *X^2^* \| *P*-value \| \| Gender \| male \| 1467（87.7%） \| 206（12.3%） \| 34.510 \| <0.001 \| \| female \| 1675（80.6%） \| 404（19.4%） \| \| Age(y) \| 65-74 \| 2058(84.7%) \| 373(15.3%) \| 8.332 \| 0.016 \| \| 75-84 \| 911(81.2%) \| 211(18.8%) \| \| ≥85 \| 173(86.9%) \| 26(13.1%) \| \| Mental  health \| likely well \| 2422（85.5%） \| 412（14.5%） \| 43.320 \| <0.001 \| \| mild mental disorder \| 453（82.5%） \| 96（17.5%） \| \| moderate mental disorder \| 151（72.2%） \| 58（27.8%） \| \| severe mental disorder \| 85（70.2%） \| 36（29.8%） \| \| ADL \| poor \| 53（81.5%） \| 12（18.5%） \| 10.901 \| 0.012 \| \| medium \| 20（74.1%） \| 7（25.9%） \| \| good \| 20（64.5%） \| 11（35.5%） \| \| very well \| 2998（84.1%） \| 566（15.9%） \| \| IADL \| poor \| 60（69.0%） \| 27（31.0%） \| 38.560 \| 0.001 \| \| medium \| 48（65.8%） \| 25（34.2%） \| \| good \| 292（80.4%） \| 71（19.6%） \| \| very well \| 2697（85.1%） \| 473（14.9） \| \| Bone density \| normal \| 465（86.1%） \| 75（13.9%） \| 6.101 \| 0.047 \| \| osteopenia \| 435（80.6%） \| 105（19.4%） \| \| osteoporosis \| 233（82.3%） \| 50（17.7%） \| \| Marital status \| with spouse \| 2149（84.4%） \| 397（15.6%） \| 8.398 \| 0.038 \| \| divorced or widowed \| 899（81.6%） \| 203（18.4%） \| \| single \| 41（93.2%） \| 3（6.8%） \| \| remarried \| 8（72.7%） \| 3（27.3%） \| \| Education level \| never attended school \| 1177（81.5%） \| 268（18.5%） \| 18.641 \| 0.001 \| \| Primary school \| 1238（83.9%） \| 237（16.1%） \| \| Junior high school \| 466（87.1%） \| 69（12.9%） \| \| High school（technical secondary） \| 154（90.1%) \| 17(9.9%) \| \| College or above \| 45(93.8%） \| 3（6.2%） \| \| Income level \| ＜5thousand \| 2128（82.3%） \| 457（17.7%） \| 17.217 \| 0.002 \| \| 5-20thousand \| 540（86.5%） \| 84（13.5%） \| \| 20-40thousand \| 300（89.8%) \| 34(10.2%) \| \| 40-60thousand \| 88(85.4%) \| 15(14.6%) \| \| ≥60thousand \| 17(89.5%) \| 2(10.5%） \| \| Source  of income \| self-paying \| 776（86.2%） \| 124（13.8%） \| 23.397 \| 0.001 \| \| retirement pension \| 598（87.3%） \| 87（12.7%） \| \| spousal burden \| 31（79.5%） \| 8（20.5%） \| \| From children \| 485（79.1%） \| 128（20.9%） \| \| government funding \| 1168（82.5%) \| 24(17.5%) \| \| from relatives and friends \| 6(100.0%) \| 0(0.0%) \| \| endowment insurance \| 53(82.8%) \| 11(17.2%) \| \| Children working outside \| all out≥6 months \| 1156（81.7%） \| 259（18.3%） \| 9.135 \| 0.010 \| \| all out≥3months \| 125（90.6%） \| 13（9.4%） \| \| ≥a child at home \| 1673（84.2%） \| 313（15.8%） \| \| Floor tiles \| non-slippery floor \| 1228（85.9%） \| 202（14.1%） \| 9.664 \| 0.008 \| \| slippery floor \| 656（84.1%） \| 124（15.9%） \| \| non-slippery floor \| 1162（81.6%） \| 262（18.4%） \| \| Height of stairs \| yes \| 2307（84.8%） \| 414（15.2%） \| 9.596 \| 0.008 \| \| no \| 696（80.5%） \| 169（19.5%) \| \| No stairs \| 18(90.0%) \| 2(10.0%） \| \| Domicile  near road \| yes \| 1590（85.3%） \| 275（14.7%） \| 6.092 \| 0.014 \| \| no \| 1473（82.2%） \| 318（17.8%） \| \| Fresh  fruits  intake frequency \| daily \| 390（86.9%） \| 59（13.1%） \| 11.125 \| 0.011 \| \| often \| 883（85.7%） \| 148（14.3%） \| \| occasionally \| 1250（82.8%） \| 259（17.2%） \| \| hardly \| 580（80.9%） \| 137（19.1%） \| \| Meat intake frequency \| daily \| 382（85.1%） \| 67（14.9%） \| 11.648 \| 0.009 \| \| often \| 1122（85.7%） \| 187（14.3%） \| \| occasionally \| 1370（82.5%） \| 291（17.5%） \| \| hardly \| 216（78.6%） \| 59（21.4） \| \| Roughage intake frequency \| daily \| 374（88.6%） \| 48（11.4%） \| 11.412 \| 0.010 \| \| often \| 866（84.3%） \| 161（15.7%） \| \| occasionally \| 1046（82.8%） \| 218（17.2%） \| \| hardly \| 775（81.7%） \| 174（18.3） \| \| Cane use \| yes \| 196（66.2%） \| 100（33.8%） \| 69.889 \| 0.001 \| \| no \| 2746（85.1%） \| 482（14.9%） \| \| Eyesight \| watch TV unclearly \| 441（80.9%） \| 104（19.1%） \| 20.119 \| 0.001 \| \| watch TV clearly \| 1754（82.9%） \| 360（17.1%） \| \| read book clearly \| 859（87.4%） \| 124（12.6%） \| \| others \| 17（65.4%） \| 9（34.6%） \| \| Sleep time \| ≤4h \| 259（83.3%） \| 59（16.7%） \| 25.112 \| 0.001 \| \| 5h \| 428（78.4%） \| 118（21.6%） \| \| 6h \| 575（81.9%） \| 127（18.1%） \| \| 7h \| 529（85.7%） \| 88（14.3%) \| \| 8h \| 643(87.8%) \| 89(12.2%) \| \| 9h \| 564(85.1%) \| 99(14.9%) \| \| Hypertension \| no \| 1669（85.1%） \| 292（14.9%） \| 7.239 \| 0.007 \| \| yes \| 1375（81.8%） \| 306（18.2%） \| \| Diabetes \| no \| 2730（84.3%） \| 509（15.7%） \| 10.596 \| 0.001 \| \| yes \| 314（77.9%） \| 89（22.1%） \| \| Heart disease \| no \| 2384（84.5%） \| 436（15.5%） \| 8.365 \| 0.004 \| \| yes \| 660（80.3%） \| 162（19.7%） \| \| Tumor \| no \| 3093（83.9%） \| 593（16.1%） \| 4.453 \| 0.035 \| \| yes \| 49（74.2%） \| 17（25.8%） \| \| Stroke \| no \| 2839（84.6%） \| 517（15.4%） \| 16.983 \| 0.001 \| \| yes \| 303（76.5%） \| 93（23.5%） \| \| Arthritis \| no \| 2545（84.7%） \| 460（15.3%） \| 10.009 \| 0.002 \| \| yes \| 597（79.9%） \| 150（20.1%） \| \| Cervical spondylosis \| no \| 2459（84.9%） \| 437（15.1%) \| 12.724 \| 0.001 \| \| yes \| 683(79.8%) \| 173(20.2%) \| \| Hepatocirrhosis \| no \| 3119（83.6%） \| 610（16.4%） \| 4.493 \| 0.034 \| \| yes \| 23（100.0%） \| 0（0.0%） \| \| Cataract \| no \| 2800（84.6%） \| 508（15.4%） \| 16.679 \| 0.001 \| \| yes \| 342（77.0%） \| 102（23.0%） \| \| Number of medicine \| 0-1 \| 1792（85.8%） \| 298（14.2%） \| 28.136 \| 0.001 \| \| 2-5 \| 984（79.7%） \| 251（20.3%） \| \| 6-7 \| 55（79.7%） \| 14（20.3%） \| \| ≥8 \| 13（61.9%） \| 8（38.1%） \| |
| --- | --- | --- | --- | --- | --- | --- | --- | --- | --- | --- | --- | --- | --- | --- | --- | --- | --- | --- | --- | --- | --- | --- | --- | --- | --- | --- | --- | --- | --- | --- | --- | --- | --- | --- | --- | --- | --- | --- | --- | --- | --- | --- | --- | --- | --- | --- | --- | --- | --- | --- | --- | --- | --- | --- | --- | --- | --- | --- | --- | --- | --- | --- | --- | --- | --- | --- | --- | --- | --- | --- | --- | --- | --- | --- | --- | --- | --- | --- | --- | --- | --- | --- | --- | --- | --- | --- | --- | --- | --- | --- | --- | --- | --- | --- | --- | --- | --- | --- | --- | --- | --- | --- | --- | --- | --- | --- | --- | --- | --- | --- | --- | --- | --- | --- | --- | --- | --- | --- | --- | --- | --- | --- | --- | --- | --- | --- | --- | --- | --- | --- | --- | --- | --- | --- | --- | --- | --- | --- | --- | --- | --- | --- | --- | --- | --- | --- | --- | --- | --- | --- | --- | --- | --- | --- | --- | --- | --- | --- | --- | --- | --- | --- | --- | --- | --- | --- | --- | --- | --- | --- | --- | --- | --- | --- | --- | --- | --- | --- | --- | --- | --- | --- | --- | --- | --- | --- | --- | --- | --- | --- | --- | --- | --- | --- | --- | --- | --- | --- | --- | --- | --- | --- | --- | --- | --- | --- | --- | --- | --- | --- | --- | --- | --- | --- | --- | --- | --- | --- | --- | --- | --- | --- | --- | --- | --- | --- | --- | --- | --- | --- | --- | --- | --- | --- | --- | --- | --- | --- | --- | --- | --- | --- | --- | --- | --- | --- | --- | --- | --- | --- | --- | --- | --- | --- | --- | --- | --- | --- | --- | --- | --- | --- | --- | --- | --- | --- | --- | --- | --- | --- | --- | --- | --- | --- | --- | --- | --- | --- | --- | --- | --- | --- | --- | --- | --- | --- | --- | --- | --- | --- | --- | --- | --- | --- | --- | --- | --- | --- | --- | --- | --- | --- | --- | --- | --- | --- | --- | --- | --- | --- | --- | --- | --- | --- | --- | --- | --- | --- | --- | --- | --- | --- | --- | --- | --- | --- | --- | --- | --- | --- | --- | --- | --- | --- | --- | --- | --- | --- | --- | --- | --- | --- | --- | --- | --- | --- | --- | --- | --- | --- | --- | --- | --- | --- | --- | --- | --- | --- | --- | --- | --- | --- | --- | --- | --- | --- | --- | --- | --- | --- | --- | --- | --- | --- | --- | --- | --- | --- | --- | --- | --- | --- | --- | --- | --- | --- | --- | --- | --- | --- | --- | --- | --- | --- | --- | --- |
